# Ruminal digestion, gastrointestinal microbial profile, and metabolic pathways after the introduction of silicon-containing ultrafine particles into bull

**DOI:** 10.14202/vetworld.2025.1070-1081

**Published:** 2025-04-30

**Authors:** Elena Sizova, Elena Yausheva, Sergey Miroshnikov, Aina Kamirova, Daniil Shoshin

**Affiliations:** 1 Scientific-educational center “Biological Systems and Nanotechnologies”, Orenburg State University, 13, prosp. Pobedy, 460018, Russia; 2 Laboratory “Agroecology of technogenic nanomaterials”, Federal Research Center of Biological Systems and Agrotechnologies, Russian Academy of Sciences, 29, ul. 9 Yanvarya, Orenburg, 460000, Russia

**Keywords:** cattle, digestibility, feed efficiency, Kyoto encyclopedia of genes and genomes, metabolic pathways, nanotechnology, *Prevotella*, rumen microbiota, silicon dioxide, ultrafine particles

## Abstract

**Background and Aim:**

Nanotechnology offers innovative strategies to enhance livestock productivity and sustainability. Silicon-containing ultrafine particles (UFPs) have shown potential benefits in animal nutrition, yet their effects on gastrointestinal microbial composition and ruminal digestion in cattle remain poorly understood. This study was to evaluate the impact of dietary supplementation with silicon-containing UFPs on ruminal digestibility, bacterial taxonomic structure, and predicted metabolic functions in the gastrointestinal microbiota of cattle.

**Materials and Methods:**

A 42-day controlled feeding experiment was conducted on 20 Kazakh white-headed bulls (12 months old, 305 ± 10.4 kg), divided into control and experimental groups (n = 10 each). The experimental group received a diet supplemented with SiO_2_ UFPs (2 mg/kg feed). Digestibility coefficients were measured using standard methods, and ruminal fluid samples were subjected to 16S ribosomal RNA sequencing and Kyoto encyclopedia of genes and genomes -based functional profiling.

**Results:**

UFP supplementation significantly increased the digestibility of dry matter (3.5%), crude fiber (3.5%), crude protein (5.2%), and organic matter (8.11%) compared to the control group. The experimental group exhibited elevated relative abundances of *Prevotellaceae*, *Lachnospiraceae*, *Oscillospiraceae*, and genera *Prevotella*, *Ruminococcus*, and *Selenomonas*. Functional prediction analysis revealed higher proportions of genes involved in carbohydrate metabolism (e.g., starch, galactose, and amino sugar pathways), lipid metabolism, oxidative phosphorylation, and the biosynthesis of key vitamins and cofactors. Microbial diversity metrics (Chao1, Shannon) indicated significant changes in alpha diversity, with moderate shifts in beta diversity.

**Conclusion:**

Dietary inclusion of silicon-containing UFPs enhances nutrient digestibility and induces favorable modifications in the ruminal microbiota, including functional pathways linked to energy and macronutrient metabolism. These findings support the integration of nanotechnology-based feed additives in cattle nutrition to improve feed efficiency, productivity, and potentially reduce environmental impacts such as methane emissions.

## INTRODUCTION

Livestock farming plays a pivotal role in global agriculture, serving as a primary source of food and raw materials for both the food and light industries. Products derived from livestock, including meat, milk, eggs, and other essential food items, are fundamental to human nutrition [1].

Enhancing the efficiency of livestock production has become an increasingly urgent challenge worldwide, particularly within the Russian agro-industrial sector. This is driven by the implementation of the national Food Security Doctrine and the growing emphasis on livestock product exports. Under current conditions, intensification of production and mitigation of the environmental burden are critical priorities in animal husbandry [2]. In contemporary practices, emphasis is placed on increasing output while simultaneously reducing ecological impact [3].

The advancement of livestock farming is closely tied to the pursuit of innovative strategies for improving productivity and product quality. One such strategy involves the use of biologically active substances in ultrafine particle (UFP) form, which have become increasingly common in animal agriculture [4, 5]. Metal-based UFPs have demonstrated beneficial effects on productivity and immune function, offering a promising alternative to conventional mineral supplements and antibiotics [6, 7]. A novel approach involving the administration of micro- and macroelements in ultrafine form at substantially lower dosages than traditional mineral feed additives has been shown to reduce physiological stress on animals while minimizing environmental impact [8].

Among these, silicon-based UFPs have attracted attention due to their inert behavior during absorption in the gastrointestinal tract and their potential applicability in livestock nutrition [9]. Positive effects have been reported for silicon-containing UFPs on nutrient digestibility, weight gain, and immunological responses in farm animals [10–12]. These particles also hold promise for use in conjunction with other elements to treat intestinal disorders [13]. However, previous studies by Guilloteau *et al*. [14] and Gong *et al*. [15] have documented adverse effects of silicon UFPs, particularly their potential to disrupt intestinal homeostasis. Despite this, investigations into the specific influence of UFPs on the gastrointestinal microbiota of livestock remain limited. A search of the PubMed database over the past decade using keywords such as “nanoparticles animals,” “nanoparticles cattle/cow/bull,” “nanoparticles chickens,” “nanoparticles poultry,” “nanoparticles fish,” and “nanoparticles pig” yielded over 70,000 results. Of these, more than 11,000 articles addressed the effects of nanoparticles on cattle; however, fewer than 3,000 studies specifically explored their impact on the microbiota of farm animals.

The gastrointestinal microbiota of livestock, particularly ruminants, represents a complex ecosystem that significantly influences the overall functionality of the host organism [16]. The ruminal microbiome is often referred to as a “hidden metabolic organ” due to its crucial role in enhancing feed conversion efficiency in cattle [17]. Microorganisms residing in the rumen facilitate the digestion and absorption of nutrients, biosynthesis of proteins, immune regulation, and maintenance of overall health [18, 19]. Previous studies [20–23] have established correlations between ruminal microbial composition and economically relevant traits, including feed efficiency, growth performance, meat marbling, milk yield, and milk quality. Alterations in the microbial structure of the rumen can lead to shifts in host metabolic processes and impact productivity outcomes [24]. Therefore, a comprehensive analysis of taxonomic data and functional profiling of the gastrointestinal microbiome is essential for evaluating animal health and optimizing nutritional strategies.

Given its distinct structure and function, the gastrointestinal microbiome is critically important for maintaining animal health. Diet is a major determinant of microbiota composition and functionality, influencing its dynamics significantly [25]. Nevertheless, limited information exists regarding the effects of silicon dioxide nanoparticle-enriched diets on the ruminal microbiome in cattle. Moreover, challenges related to nutrient bioavailability constrain the broader application of such nanoparticles in animal nutrition. While a previous study by Diao *et al*. [26] highlights the potential risks associated with dietary silicon dioxide UFPs, particularly in terms of gut health and microbiome stability, further investigation is required. Understanding the relationship between the functional characteristics of the gut microbiota and the digestibility of dietary components in response to nano-sized micro- and macroelement supplementation remains a critical area of research for enhancing feeding systems in cattle.

Given the growing interest in the application of nanotechnology in livestock nutrition and the limited understanding of its effects on the gastrointestinal microbiome, this study aimed to evaluate the impact of dietary supplementation with silicon-containing UFPs on ruminal digestion, microbial taxonomic composition, and predicted metabolic functions in Kazakh white-headed bulls. Specifically, the research sought to determine whether silicon UFPs could enhance nutrient digestibility and induce beneficial shifts in the structure and functionality of the ruminal microbiota, thereby contributing to improved feed efficiency and overall animal productivity.

## MATERIALS AND METHODS

### Ethical approval

All procedures involving animals were conducted in accordance with the guidelines and regulations set forth in the Model Laws of the Interparliamentary Assembly of Member States of the Commonwealth of Independent States (“On the Treatment of Animals,” Article 20) and the Guidelines for Working with Laboratory Animals of Orenburg State University. The study protocol was approved by the Ethics Committee of the Federal State Budgetary Educational Institution of Higher Education “Orenburg State University” (Protocol No. 2, dated May 15, 2024). This study followed the ARRIVE guidelines for *in vivo* study.

### Study period and location

The study was conducted in May 2024 at Orenburg State University, Russia.

### Experimental animals

The study was carried out on Kazakh white-headed bulls aged 12 months, with an average body weight of 305 ± 10.4 kg, each equipped with a ruminal fistula. This breed is one of the most commonly raised beef cattle breeds in the region. Ruminal fistulas (diameter: 80 mm; Ankom Technology Corp., USA) were surgically installed following the method described by Aliev [27]. Using the analog-pair method, the animals were divided into two groups (n = 10 each): Group I (control) received a basic diet (BD), while Group II received the same BD supplemented with SiO_2_ UFPs at a dosage of 2 mg/kg of feed. The BD was formulated according to standard feeding recommendations [28], and its composition is detailed in Table 1. The animals were fed a combined diet.

**Table 1:** Ingredients and mineral compositions of the diet.

Ingredients	Amount per head per day	Mineral compositions	Amount per head per day
Cereal-legume hay (kg)	4.5	Ca (g)	56.9
Corn silage (kg)	10	P (g)	28.6
Root crops (kg)	6.5	Mg (g)	20.4
Concentrates (kg)	3	K (g)	62
Feed phosphate (g)	50	Co (g)	5.2
Salt (g)	40	Cu (g)	69.4
		Fe (g)	580.9
		I (g)	2.64
		Mn (g)	430
		Zn (g)	388.2
		S (g)	26.7
		Al (mg)	54.6
		As (mg)	0.84
		B (mg)	52.1
		Cd (mg)	0.106
		Cr (mg)	2.41
		Hg (mg)	0.01
		Ni (mg)	1.35
		Pb (mg)	0.32
		Se (mg)	0.66
		Sn (mg)	0.11
		Sr (mg)	4.39
		V (mg)	0.11

Nutritional value of the diet: Energy feed units – 10.1; Exchange energy – 97.2 MJ; Dry matter – 10.4 kg; Crude protein – 1336 g; Digestible protein – 929 g; Crude fiber – 2075 g; Starch – 1188.5 g; Sugar – 893.8 g; Crude fat – 282.2 g

The ultrafine silicon dioxide particles used in the study were chemically pure, with a hydrodynamic diameter of 256.2 ± 10.0 nm and a zeta potential of 60.9 ± 0.5 mV. These particles were synthesized by chemical deposition in the Nanostructure Synthesis Laboratory of Orenburg State University. Before being added to the feed, the nanoparticles were subjected to ultrasonic treatment at 35 kHz, 300–450 W, and a 10 µm amplitude for 30 min to ensure dispersion.

All animals were clinically healthy, housed in individual pens (dimensions: 100 × 180 cm), kept tied, and fed individually. Water was freely available through automatic drinkers. The duration of the experiment was 42 days. Nutrient digestibility was assessed through a preparatory period (14 days) followed by a 5-day data collection period, during which composite fecal samples (representing 10% of the total daily output) were collected 3 times daily (morning, afternoon, evening). Feed intake was recorded daily throughout the digestibility trial [29].

### Determination of chemical and elemental composition

The chemical analysis of feces and feed samples was performed in triplicate at the Testing Center of the Central Collective Use Center of the Federal Research Center BST RAS (http://tskp-bst.rf, Orenburg, Russia). The following parameters were determined: Dry matter, crude protein, crude fat, crude fiber, and crude ash content [30–34]. Organic matter was calculated based on weight loss after ashing. Nitrogen-free extract was calculated by subtracting the sum of crude protein, crude fat, crude fiber, and ash from the dry matter. Elemental composition of the diet was analyzed using an inductively coupled plasma mass spectrometer (Agilent Technologies-12, USA).

### Determination of the bacterial composition of rumen fluid

Rumen fluid samples were collected from three animals in each group through the ruminal fistula under sterile conditions. Samples were preserved in DNA/RNA Shield (USA) and immediately frozen.

DNA extraction, library preparation, sequencing, and bioinformatic processing were conducted at the Center for Collective Use of Scientific Equipment “Persistence of Microorganisms,” Institute of Cellular and Intracellular Symbiosis, Ural Branch of the Russian Academy of Sciences (Orenburg, Russia). Total DNA was extracted using a combined protocol involving mechanical homogenization with a Y lysis matrix (MP Biomedicals, USA) in an LT analyzer (Qiagen, Germany) and the QIAamp Fast DNA Stool Mini Kit (Qiagen, Germany). DNA purity and concentration were assessed through NanoDrop 8000 spectrophotometry (Thermo Fisher Scientific Inc., USA) and Qubit 4 fluorometry (Life Technologies, USA), using a high-sensitivity dsDNA analysis kit.

DNA libraries were purified with Agencourt AMPure XP beads (Beckman Coulter, USA) and quality-checked using capillary electrophoresis on the QIAxcel system (Qiagen, Hilden, Germany) with the QIAxcel DNA Screening Kit. Sequencing was performed on the Illumina MiSeq platform using the MiSeq Reagent Kit V3 (2 × 300 bp, Illumina, USA).

Functional analysis of rumen microorganisms was conducted using the Kyoto encyclopedia of genes and genomes (KEGG) through MicrobiomeAnalyst, employing both the Marker Data Profiling module (Tax4Fun) and Shotgun Data Profiling module (Functional Profiling: Association Analysis).

### Statistical analysis

Statistical analysis was conducted using Statistica 10 (StatSoft, USA) to ensure the reliability and validity of the results. Descriptive statistics, including means and standard deviations, were calculated for each parameter. The Mann–Whitney U-test was used to compare differences between the control and experimental groups, as it is a robust non-parametric test suitable for independent samples. Statistical significance was defined as p ≤ 0.05.

Microbial alpha diversity was assessed using the Chao1, Shannon, and Simpson indices to evaluate richness, evenness, and overall diversity. Differences in diversity indices were tested using analysis of variance. Beta diversity analysis was performed using non-metric multidimensional scaling based on Bray–Curtis dissimilarity indices and group differences were evaluated using Permutational Multivariate Analysis of Variance.

Spearman’s rank correlation was employed to identify associations between microbial taxa and nutrient digestibility, as this method is appropriate for non-linear and ordinal data. Operational taxonomic units (OTUs) were filtered and assigned taxonomic identities for downstream analyses. Functional profiling was performed using the KEGG database through MicrobiomeAnalyst. p-values for differences in predicted metabolic pathways were adjusted using the false discovery rate method to control for multiple comparisons.

These statistical approaches ensured robust and unbiased comparisons of digestibility data, microbial composition, and predicted microbial functions between the experimental and control groups.

## RESULTS

### Nutrient digestibility coefficients

**Figure 1:**
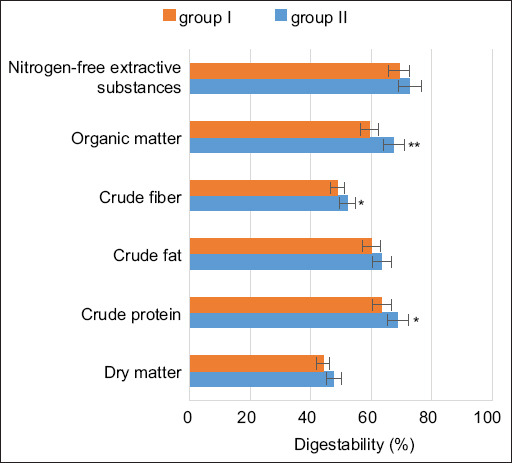
Digestibility coefficients of diet nutrients in the control and experimental groups. *p ≤ 0.05; **p ≤ 0.01 compared with the control.

Analysis of nutrient digestibility revealed that bulls in the experimental group exhibited increased digestibility coefficients compared to the control group: Dry matter (3.5%), crude fiber (3.5%), crude protein (5.2%), and organic matter (8.11%) (Figure 1). In addition, the digestibility coefficients of nitrogen-free extract and crude fat were also elevated in the experimental group relative to the control.

### Structure of the ruminal microbiome

**Figure 2:**
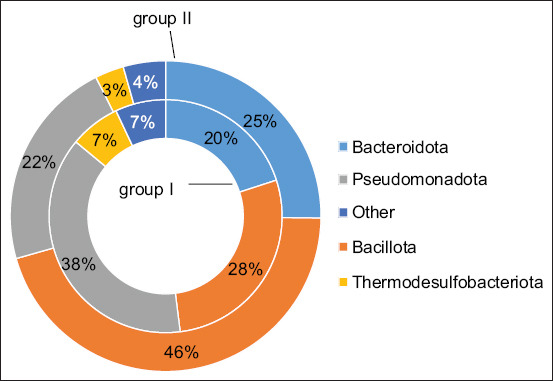
Abundance of taxonomic groups (at the phylum level) in the ruminal microbiome in the control and experimental groups. Other – this group includes taxa, the number of which does not exceed 2% of the total number of bacteria; *p ≤ 0.05, when comparing the experimental group with the control group.

Evaluation of the bacterial composition of the ruminal microbiota in the control group indicated that the predominant phyla were *Bacteroidota, Bacillota*, and *Pseudomonadota* (Figure 2). At the family level, the most abundant taxa included P*revotellaceae, Moraxellaceae, Lachnospiraceae*, and *Oscillospiraceae*, each comprising between 7% and 15.3% of the total bacterial population.

**Figure 3:**
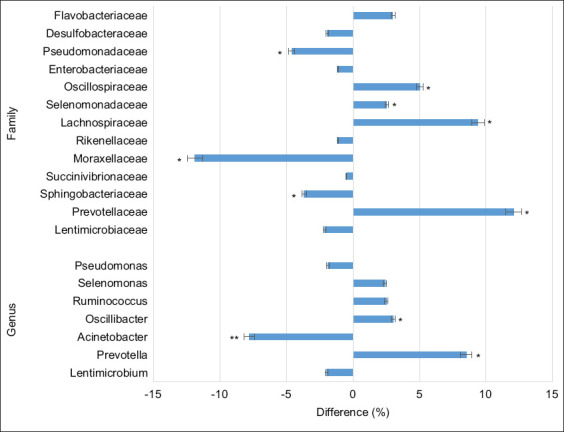
Difference in the taxonomic composition (at the family and genus level) of ruminal fluid between the experimental and control groups. *p ≤ 0.05, when comparing the experimental group with the control group.

Supplementation with silicon dioxide UFPs led to marked shifts in microbial composition within the rumen of the experimental group. Specifically, there was an increased relative abundance of bacteria from the phyla *Bacteroidota* (p = 0.038) and *Bacillota* (p = 0.041). At the family level, the experimental group exhibited significantly higher proportions of *Prevotellaceae* (p = 0.035), *Lachnospiraceae* (p = 0.018), *Oscillospiraceae* (p = 0.027), *Selenomonadaceae*, and *Flavobacteriaceae* compared with the control (Figure 3).

At the genus level, notable increases were observed in *Prevotella* (p = 0.042), *Oscillibacter* (p = 0.037), *Ruminococcus* (p = 0.031), and *Selenomonas* (p = 0.025) in the experimental group. Conversely, reductions were recorded in *Moraxellaceae* (11.9%, p ≤ 0.05), *Pseudomonadaceae* (4.6%, p ≤ 0.05), and *Acinetobacter* (7.8%, p ≤ 0.01) relative to the control group.

### Rumen microbial diversity

**Figure 4:**
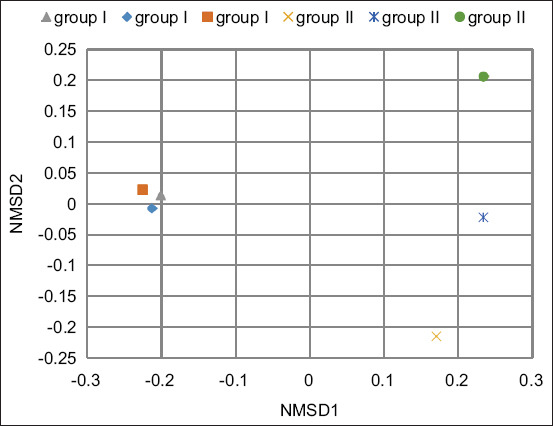
Ruminal microbial beta diversity in experimental groups using permutational multivariate analysis of variance statistical method, nonmetric multivariate scaling and Bray–Curtis dissimilarity.

Analysis of microbial diversity, which included assessments of richness, evenness, and homogeneity, revealed a significantly higher Chao1 index in the experimental group compared to the control (Table 2). Beta diversity analysis further demonstrated differences in bacterial community organization between the two groups (p = 0.1) (Figure 4).

**Table 2:** Indices of species diversity in ruminal microbiota of bulls after using silicon-containing.

Indicator	Group I	Group II	p-value
Chao1	207.6 ± 1.86	264 ± 6.03	0.007
Simpson	0.963 ± 0.003	0.946 ± 0.003	0.015
Shannon	4.087 ± 0.039	3.863 ± 0.014	0.021

Spearman’s rank correlation analysis revealed significant positive associations between crude protein digestibility and the taxa *Oscillospiraceae, Ruminococcus, Selenomonas*, and *Oscillibacter* in the ruminal microbiota (Table 3). Likewise, crude fiber digestibility was strongly correlated with the abundance of *Oscillospiraceae*. Positive correlations were also identified between crude fat digestibility and *Pseudomonadaceae*, as well as between nitrogen-free extract digestibility and the genera *Prevotella* and *Selenomonas*. In contrast, negative correlations were observed between crude protein digestibility and the families *Lentimicrobiaceae* and *Pseudomonadaceae*.

**Table 3:** Spearman rank correlation was used to assess the close relationship between nutrient digestibility coefficients and the taxonomic diversity of the ruminal microbiome in animals of the experimental group.

Indicators	Dry matter	Crude protein	Crude fat	Crude fiber	Organic matter	Nitrogen-free substances
*Oscillospiraceae*	−0.085	**0.828**	−0.211	0.641	0.428	0.086
*Pseudomonadaceae*	0.144	**−0.841**	0.588	−0.029	−0.462	−0.1741
*Lentimicrobium*	−0.600	**−0.886**	0.579	0.371	−0.514	−0.4864
*Prevotella*	0.028	0.600	−0.551	−0.257	0.371	0.600
*Oscillibacter*	0.257	**0.714**	−0.241	0.028	0.486	0.371
*Ruminococcus*	−0.085	**0.885**	−0.493	0.628	0.543	0.143
*Selenomonas*	0.257	**0.886**	−0.493	0.371	0.486	0.543

Significant correlations at the p ≤ 0.05 level are marked in bold.

### Predicted metabolic pathways

**Figure 5:**
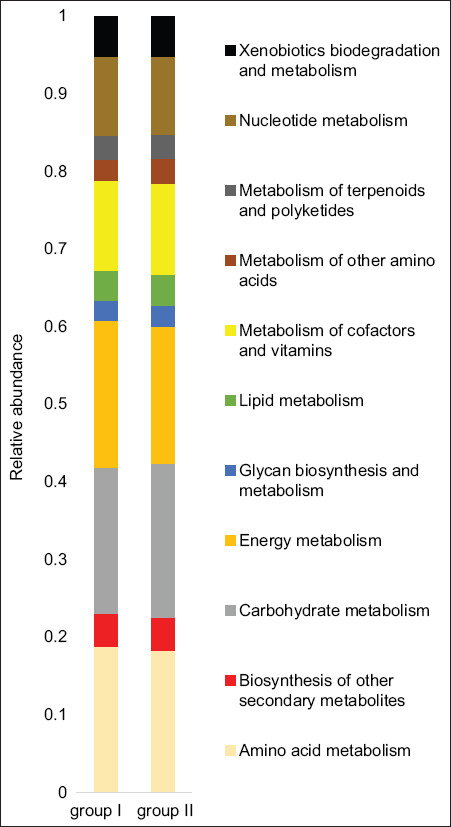
Profiling of the functional diversity of the ruminal microbiota in the animals of the experimental and control groups.

Functional profiling of the ruminal microbiota was conducted by comparing OTUs to the KEGG database (Figure 5). The major predicted pathways in both control and experimental groups were related to amino acid metabolism, cofactor and vitamin metabolism, energy metabolism, and carbohydrate metabolism.

**Figure 6:**
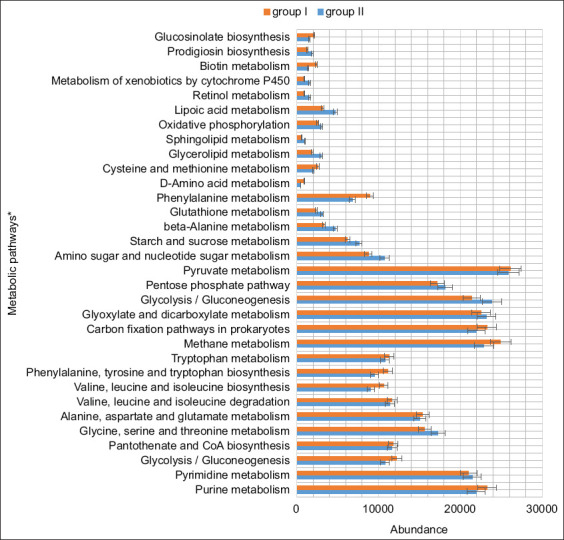
Number of microbial phenotypes predicting metabolic pathways in the control and experimental groups. *Indicates metabolic pathways with a significance threshold of p < 0.01.

In the control group, there was high gene abundance associated with pyruvate metabolism, as well as pathways linked to glyoxylate and dicarboxylate metabolism, glycolysis/gluconeogenesis, and the pentose phosphate pathway (Figure 6). High proportions of genes involved in methane metabolism and carbon fixation in prokaryotes were also detected. Amino acid metabolism in this group prominently featured pathways for glycine, serine, and threonine metabolism; alanine, aspartate, and glutamate metabolism; and the biosynthesis/degradation of valine, leucine, isoleucine, phenylalanine, tyrosine, and tryptophan. Cofactor and vitamin metabolism was characterized by high abundance of genes related to porphyrin metabolism, pantothenate and CoA biosynthesis, and folate biosynthesis. The metabolism of purines and pyrimidines constituted the dominant nucleotide metabolic pathways.

In the experimental group, these core metabolic pathways were similarly predominant. However, gene abundance was significantly higher for pathways associated with starch and sucrose metabolism, amino sugar and nucleotide sugar metabolism (p = 6.459e-05), and galactose metabolism (p = 5.499e-05). Within amino acid metabolism, a greater abundance of genes involved in beta-alanine (p = 7.654e-06) and glutathione metabolism (p = 1.129e-05) was detected compared to the control. Conversely, genes associated with phenylalanine metabolism (p = 2.078e-05), D-amino acid metabolism (p = 1.066e-05), and cysteine and methionine metabolism (p = 3.912e-05) were less abundant in the experimental group.

The experimental group also exhibited higher gene abundance in pathways associated with glycerolipid metabolism (p = 0.007) and sphingolipid metabolism (p = 1.933e-05). Regarding energy metabolism, an increased abundance of genes related to oxidative phosphorylation (p = 0.004) was observed. Moreover, genes linked to the metabolism of retinol (p = 1.929e-05), lipoic acid (p = 0.0002), and cytochrome P450-mediated xenobiotic metabolism (p = 1.929e-05) were more prevalent, while biotin metabolism genes were less abundant (p = 0.001). Functional gene profiles also revealed elevated biosynthetic activity related to glucosinolates (p = 3.243e-05) and prodigiosin (p = 0.001) in the experimental group.

Overall, the results demonstrated that dietary inclusion of ultrafine silicon dioxide particles enhanced nutrient digestibility and altered the taxonomic and functional structure of the rumen microbiome. These microbial shifts contributed to increased abundance of genes associated with carbohydrate metabolism, which may have beneficial implications for animal health and reduced methane emissions.

## DISCUSSION

Feed additives containing UFPs are widely utilized in livestock production, including in the diets of cattle [35, 36]. Previous studies [37–40] have reported the beneficial effects of UFP supplementation on nutrient digestibility and productivity traits in farm animals, such as improved milk quality and increased live weight gain. However, several investigations have also highlighted potential adverse outcomes associated with UFP use in animal husbandry. Negative effects documented include weight loss, organ pathology, and increased mortality rates [41, 42]. The chronic ingestion of nanoform trace elements has been associated with inflammatory changes in the intestinal epithelium and disturbances in the balance of the intestinal microflora [43]. Importantly, the occurrence of such pathological effects has been shown to be dose-dependent [44].

Given the growing recognition of the central role of the gastrointestinal microbiota in animal health, the potential negative impact of nanoparticles on microbial ecosystems warrants close scrutiny. However, the influence of UFPs on gastrointestinal microbiota composition and function – particularly in the context of nutrient absorption – remains insufficiently investigated. Shifts in the taxonomic structure of the gastrointestinal microbiome in response to UFP exposure may induce functional alterations, which in turn can lead to either beneficial or detrimental effects on animal physiology [45]. A strong link has been established between rumen microbial composition and feed efficiency [46]. Consequently, optimizing diets for enhanced productivity requires a detailed understanding of the ruminal microbial community, which is integral to improving meat and milk production systems.

In the present study, dietary supplementation with silicon-containing UFPs was associated with an increased Bacillota/Bacteroidota ratio in the ruminal microbiota, along with elevated abundances of *Prevotellaceae* (*Prevotella*), *Lachnospiraceae, Oscillospiraceae* (*Oscillibacter* and *Ruminococcus*), and *Selenomonadaceae* (*Selenomonas*). These microbial shifts corresponded with a higher prevalence of bacteria known to produce short-chain fatty acids (SCFAs), which are beneficial for host energy metabolism and gut health [47, 48].

The Bacillota/Bacteroidota ratio in the human gut microbiome has been linked to increases in body mass index (BMI) [49]. In cattle, a positive correlation has been observed between the abundance of Bacillota – especially *Lachnospiraceae* – and increased milk fat yield, while *Prevotella* abundance has been associated with intramuscular fat content, and *Selenomonas* with higher live weight [50–52]. Furthermore, *Lachnospiraceae* and *Oscillospiraceae* are typically more abundant in the rumen of cattle with higher feed intake, and their presence correlates with improved cellulose digestibility [53]. There is also a broader association between the dominance of Bacillota taxa and improved feed conversion efficiency. Previous studies [54–56] have reported that cattle with rumen microbiomes enriched in *Prevotellaceae, Lachnospiraceae*, and *Oscillospiraceae* exhibit superior feed efficiency metrics.

In agreement with the literature, the present study identified a close association between increased digestibility of crude protein and organic matter and elevated levels of *Selenomonas* and *Ruminococcus*. An improvement in crude fiber digestibility was also observed in the experimental group relative to the control, likely attributable to the increased abundance of *Oscillospiraceae*. These bacteria contribute to more effective fiber fermentation, thereby enhancing nutrient utilization.

Microorganisms belonging to the genus *Ruminococcus* are recognized as primary cellulolytic bacteria that actively break down fiber into simpler saccharides [57]. Likewise, higher proportions of *Oscillospiraceae* are associated with increased cellulase activity in the rumen, promoting better utilization of fibrous feedstuffs [58]. Correlation analysis from the present study revealed a moderate positive relationship (r = 0.628) between *Ruminococcus* abundance and crude fiber digestibility.

Functional predictions further revealed that, in the experimental group, gene abundance related to the metabolism of amino sugars, nucleotides, starch, sucrose, and galactose was elevated compared to the control group. These findings align with reports indicating that ruminal microbiomes in feed-efficient cattle are enriched in pathways associated with carbohydrate metabolism involving mono-, di-, and oligosaccharides [22]. A parallel increase in the expression of enzymes involved in fiber degradation was also observed, suggesting improved metabolic potential for dietary fiber utilization [59].

In addition, an upregulation of genes associated with β-alanine and glutathione metabolism was observed in the experimental group. β-alanine synthesis pathways have been linked to enhanced microbial protein production and improved digestibility of dry and organic matter [60, 61]. *Prevotellaceae* and *Ruminococcus_2* are taxa commonly associated with both amino acid and carbohydrate metabolism [62]. Li *et al*. [63] have also shown that enriched rumen pathways related to alanine, arginine, and proline metabolism are positively correlated with the abundance of *Prevotella* and *Ruminobacter*. A positive association has also been documented between *Prevotella* and metabolic pathways related to glutathione, starch, sucrose, and galactose metabolism [21].

Regarding energy metabolism, both experimental and control groups showed pyruvate metabolism as a dominant pathway. However, gene abundance for glycerolipid metabolism was higher in the experimental group. Glycerolipid metabolism is essential for cellular energy supply and plays a critical role in supporting lactation [64].

Notably, an increased abundance of genes involved in the metabolism of cofactors and vitamins was recorded following UFP supplementation. In particular, elevated gene expression was observed for the biosynthesis of prodigiosin – an antimicrobial compound – and for retinol metabolism, which is known to influence immune function and the development of critical physiological systems [65, 66]. Given the positive effects of retinol on reproductive health and embryonic development in cattle, these findings suggest that silicon-containing UFPs may have broader applications in enhancing animal health and reproductive performance [67].

## CONCLUSION

This study provides compelling evidence that dietary supplementation with silicon-containing UFPs significantly enhances nutrient digestibility and beneficially alters the ruminal microbiota in Kazakh white-headed bulls. Specifically, the experimental group demonstrated improved digestibility coefficients for dry matter (3.5%), crude protein (5.2%), crude fiber (3.5%), and organic matter (8.11%) compared to controls. These physiological improvements were accompanied by pronounced shifts in the ruminal microbial community, characterized by increased abundances of functionally significant taxa such as *Prevotella*, *Ruminococcus*, *Oscillibacter*, and *Selenomonas*. Functional predictions based on KEGG pathway analysis revealed enrichment in genes associated with carbohydrate metabolism, oxidative phosphorylation, and the biosynthesis of vitamins and cofactors, suggesting enhanced microbial efficiency and host metabolic potential.

The strength of this study lies in its integrative approach, combining *in vivo* digestibility trials with 16S ribosomal RNA sequencing and predictive functional profiling. This methodological synergy provides comprehensive insights into how silicon UFPs modulate both physiological and microbial parameters relevant to animal nutrition and health.

However, certain limitations must be acknowledged. The study’s duration (42 days) was relatively short, potentially limiting the evaluation of long-term physiological or ecological impacts of UFP supplementation. The experiment was also restricted to a single cattle breed under controlled conditions, which may constrain the generalizability of the findings across other breeds, feeding systems, or environmental contexts.

Future research should extend these findings by evaluating the chronic effects of UFPs on animal performance, gut health, immune response, and reproductive traits under commercial farming conditions. Furthermore, multi-omics analyses, including metagenomics, transcriptomics, and metabolomics, are recommended to elucidate the precise molecular mechanisms by which UFPs influence microbial functionality and host physiology. Assessing potential environmental impacts, such as changes in methane emissions or excreted nanoparticle residues, would also contribute to a more holistic evaluation of UFPs as sustainable feed additives.

This study supports the use of silicon-containing UFPs as a promising nutritional strategy for improving feed efficiency, animal productivity, and microbial health in ruminants. The incorporation of nanotechnology into livestock diets represents a novel avenue for optimizing animal agriculture in line with sustainability and precision nutrition goals.

## AUTHORS’ CONTRIBUTIONS

ES and SM: Conceptualization, methodology, and data curation. DS and AK: Validation and formal analysis. EY, DS, and AK: Investigated and collected the samples. ES, EY, and SM: Drafted, reviewed, and edited the manuscript. All authors have read and agreed to the published version of the manuscript. All authors have read and approved the final manuscript.
